# Dynamic Patterns of Expertise: The Case of Orthopedic Medical Diagnosis

**DOI:** 10.1371/journal.pone.0158820

**Published:** 2016-07-14

**Authors:** Dan Assaf, Eyal Amar, Norbert Marwan, Yair Neuman, Moshe Salai, Ehud Rath

**Affiliations:** 1 Sackler Faculty of Medicine, Tel Aviv University, Tel Aviv, Israel; 2 Orthopedics Division, Tel Aviv Sourasky Medical Center, 6 Weizmann Street, Tel Aviv, Israel; 3 Potsdam Institute for Climate Impact Research, Potsdam, Germany; 4 Department of Education, Homeland Security Institute, Center for the Study of Conversion and Inter-Religious Encounters, Ben-Gurion University of the Negev, Beer-Sheva, Israel; Tianjin University, CHINA

## Abstract

The aim of this study was to analyze dynamic patterns for scanning femoroacetabular impingement (FAI) radiographs in orthopedics, in order to better understand the nature of expertise in radiography. Seven orthopedics residents with at least two years of expertise and seven board-certified orthopedists participated in the study. The participants were asked to diagnose 15 anteroposterior (AP) pelvis radiographs of 15 surgical patients, diagnosed with FAI syndrome. Eye tracking data were recorded using the SMI desk-mounted tracker and were analyzed using advanced measures and methodologies, mainly recurrence quantification analysis. The expert orthopedists presented a less predictable pattern of scanning the radiographs although there was no difference between experts and non-experts in the deterministic nature of their scan path. In addition, the experts presented a higher percentage of correct areas of focus and more quickly made their first comparison between symmetric regions of the pelvis. We contribute to the understanding of experts’ process of diagnosis by showing that experts are qualitatively different from residents in their scanning patterns. The dynamic pattern of scanning that characterizes the experts was found to have a more complex and less predictable signature, meaning that experts’ scanning is simultaneously both structured (i.e. deterministic) and unpredictable.

## Introduction

Medical diagnosis is an “artful” skill that takes many years to master. One specific aspect of medical diagnosis involves the interpretation of radiographs. While student physicians are taught to analyze radiographs according to well-defined protocols, it is clear that experience plays a major role in the correct interpretation of radiographs (e.g., [1]), as manifested, for instance, in the superior diagnostic accuracy of expert radiologists over residents (e.g., [2]). However, beyond measures of performance (e.g., accuracy) in which experts are trivially expected to perform better than non-expert physicians, we are far from fully understanding the exact processes underlying this expertise. Studying the process of learning the diagnostic expertise should involve a longitudinal study where the declarative and procedural knowledge of the non-experts is measured along a timeline and the transformations of these knowledge structures are closely analyzed. While the learning process underlying the emergence of expertise is quite elusive, and studying it is beyond the scope of the current paper, we may try and better understand the nature of expertise by pinpointing previously unidentified aspects of this specific expertise in the interpretation of radiographs through the comparison of experts and non-experts.

In this context, the eye tracker is a powerful tool for studying radiography expertise as it allows us to identify and analyze the radiologist’s [1] focus of attention and [2] patterns of scanning the radiograph. Using eye tracking, several studies [3–5], have identified significant differences between experts and non-expert in scanning a radiograph such as fewer fixations per image, faster scanning time to diagnosis, and shorter time spent on areas of interest (AOIs).

The major aim of the current study is to analyze differences between experts’ and residents’ dynamic patterns for scanning radiographs. That is, the major aim of our study is to focus on the dynamic aspect of the diagnosis by analyzing the scan path trajectories of experts and non-experts. Our study design focuses on a specific medical problem, which is femoroacetabular impingement (FAI). FAI is a relatively newly identified and complex patho-mechanical syndrome in which joint damage may occur as a result of pathological contact stresses and/or may be associated with hip osteoarthritis (e.g., [6]). Radiographic analysis of anteroposterior (AP) pelvic x-ray is an important phase in the diagnosis of FAI (e.g., [7]), and it may take several years for physicians to gain expertise in this technique. Therefore, this study’s focus on FAI is fully justified given both the importance of interpreting a radiograph in diagnosing FAI and the long period of time it takes to gain expertise in this diagnosis technique.

**Fig 1:**
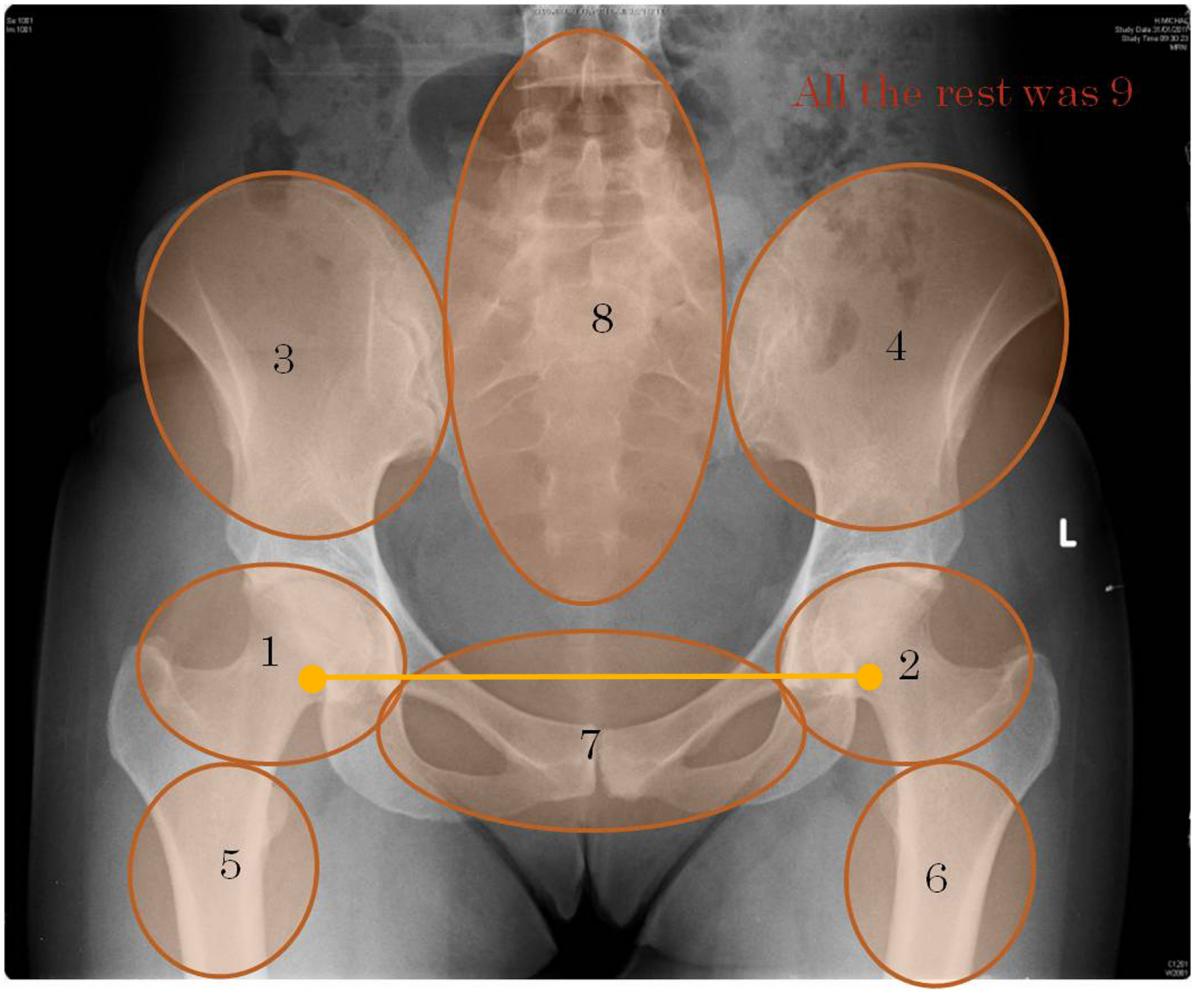
A comparison and RQA areas example. A radiograph with a comparison marked by a yellow line. This comparison represents a saccadic movement between two fixations in opposite symmetric areas. The radiograph also represent eight highlighted FOI areas where the unmarked area is considered as the ninth area. These areas were used for the RQA.

We generated three hypotheses concerning the expected differences between expert and non-expert diagnosis patterns:

1. The scan path hypothesis: Experts in scanning radiographs do not use a simple scan path where the eyes trivially move along an expected set of points. Otherwise, learning how to be an expert radiologist would be an easy task. Therefore, we hypothesized that experts would express a more complex and "chaotic" (i.e. deterministic albeit unexpected) pattern for scanning the image and moving between AOIs. This is the main and most important hypothesis of our study.
2. The AOF hypothesis: areas of interest (AOIs) are the loci where the pathology appears in a radiograph and areas of focus (AOFs) are the loci where subjects focus their attention. It has been argued [8] that experts tend to fixate more on the pathological areas while making fewer fixations overall on each radiograph. Therefore, it is reasonable to hypothesize that experts would have fewer fixation areas but with a higher ratio of AOFs to AOIs. This hypothesis explains expertise as involving a scanning pattern in which the eyes’ trajectory visits pathological areas, which function as its “attractors.”
3. The comparison hypothesis: Comparison between two symmetric areas (Fig 1) is an important tool in pelvic radiograph interpretation, and other forms of orthopedic diagnosis, as it allows the physician to diagnose abnormality in structural aspects of the joints. It has been shown [9] that experts are quicker at identifying pathological areas. Therefore, we hypothesized that experts would perform the first comparison between the symmetric areas of the pelvis faster than residents. This would mean that the scan path of experts would involve a well-defined trajectory along symmetric regions. This is the most specific hypothesis of our study as it uniquely concerns the diagnosis of FAI.

## Method

All experimental protocols were approved by the hospital’s ethics committee (the Helsinki Committee at the Sourasky Medical Center, Tel Aviv) due to the ethical issues involved in the analysis of patient radiographs, and all trials were performed in accordance with relevant guidelines and regulations.

### Participants

Fourteen male participants with a mean age of 44 and with an accumulated 107 years of experience (range 2–30 years; mean 7.62 years) volunteered to take part in the study. The participants were seven orthopedics residents with at least two years of experience and seven board-certified orthopedists. All participants gave their informed consent verbally before the experiment. The physicians who volunteered to participate in the research, both experts and novices, were part of the orthopedics department’s staff. As there was no risk in the research and as the subjects were not anonymous to the researcher, consent was given verbally and informally and was registered by the experimenter on a checklist near the participant’s name and background information.

### Stimuli

The stimuli consisted of AP pelvis radiographs. Fifteen radiographs of patients with FAI injuries (one radiograph per patient) were randomly selected from the dataset of the orthopedics department. All of the patients underwent surgical intervention due to FAI diagnosis and were surgically validated to have an FAI injury. The images were standardized in size to fit the screen resolution and all patient information was removed.

### Procedure

We used the SMI RED remote eye tracking device to record eye movements during the experiment. The system has a sampling rate of 250 Hz and a gaze accuracy of 0.4°. The screen resolution was 1680 × 1050 pixels and the screen size was 22″. The experimenter verbally explained to the participants that they would be presented with 15 AP radiographs from various cases and that they should identify the pathological evidence present on the radiograph and provide their diagnosis. The participants were asked to analyze each of the 15 radiographs and then, when they had reached their diagnosis about a certain radiograph, to state “done.” Only then were they allowed to write their diagnosis down. They were subsequently exposed to the next radiograph. On average each radiograph was scanned for 33.4 seconds (range 9–102 seconds).

Subjects were seated approximately 50 cm from the computer screen, and eye calibration and validation were performed before the experiment. Each subject was presented with the 15 slides in a random order. The subjects’ eye tracking data were collected through the SMI eye tracking device during each trial and the data were used for the analysis (the median data length was 99, where time series with length < 40 were excluded).

### Measures

The independent variable was the subject’s level of expertise (experts vs. residents). To replicate previous findings, we first used the following dependent variables:

1. slide dwell time (the time a specific slide was scanned before the participant reached a diagnosis);
2. fixation number (number of instances when the eye remained still over a period of time, usually > 200 ms);
3. fixation dwell time (the fixation length in seconds);
4. saccadic amplitude (the average distance traveled by the eye between two fixations);
5. the scan path length (the sum of the saccadic amplitudes).

### Hypotheses

#### The scan path hypothesis

**Fig 2:**
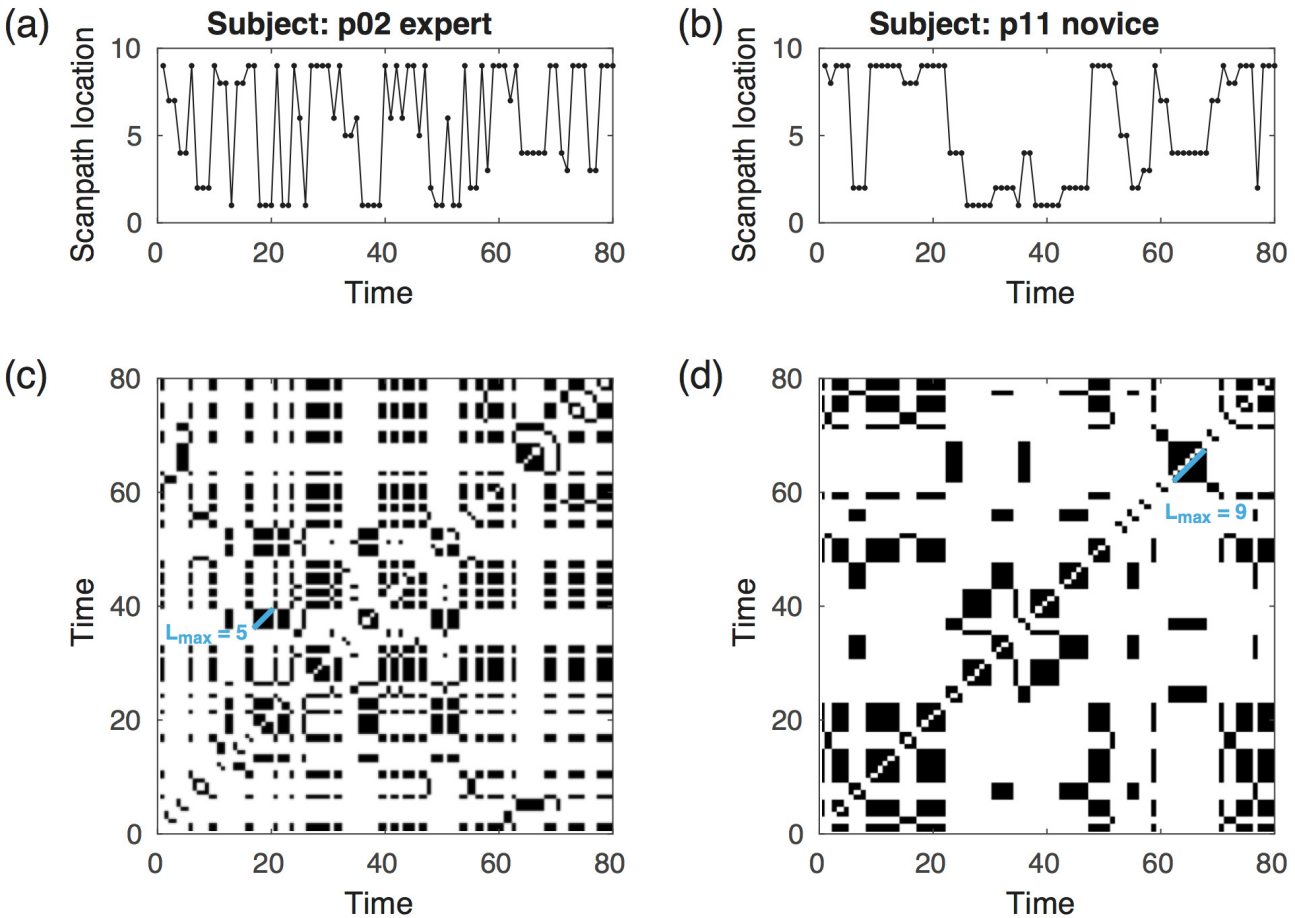
Time series and recurrence plot by expertise. Fig 2 a and b present representative time series of an expert and a novice and Fig 2c and d present the corresponding recurrence plots.

To test the scan path hypothesis, each radiograph was divided into nine AOIs (Fig 1). The boundaries of each region were arbitrarily determined by the members of the authorial team who are experts in orthopedics. We measured the fixations on these areas along a time line. The scan path of each participant was represented as a discrete time series (consisting of natural numbers between 1 and 9) of the visited areas (Fig 2a and 2b).

We analyzed these time series by using an advanced and powerful technique from nonlinear time series analysis that investigates the recurrence properties of the considered system. This technique is called recurrence quantification analysis (RQA) [10–11] and is based on the quantification of recurrence plots (RPs) [12]. An RP is a representation that visualizes the recurrences (in terms of an ε-neighborhood; see appendix) of a “path” of a dynamic system that is defined by the system’s states, i.e. the AOI in our case (Fig 2c and 2d). In general, the states analyzed by RPs are in high-dimensional state space. In our study we calculated the RPs from the time series directly without any preceding state space reconstruction.

RQA consists of several measures of complexity that focus on different recurrence aspects. For our study we selected two measures that quantify, on the one hand, the dynamic divergence behavior and, on the other hand, the "deterministic" properties of the considered system (where "deterministic" does not mean deterministic in the rigorous mathematical sense but in the sense of "non-random", "ordered" or "auto-correlated"). The divergence of the system’s states is encoded in the length of diagonal lines in the RP (Fig 2). The length of the longest line, quantified by the measure L_max_, is related to the divergence of the system, allowing conclusions to be drawn about the system’s dynamics: the shorter the longest line, the more chaotic and less predictable the system [10]. It must be emphasized that a chaotic dynamic should not be mistakenly confused with a random, “arbitrary” pattern. Chaotic dynamics are deterministic albeit unpredictable and highly sensitive to initial conditions. In the context of the scan path analysis, diagonal line segments in the RP correspond to re-used scan paths; thus, L_max_ corresponds to the longest length of a specific scan path that has been used multiple times and quantifies the predictability of the scan paths (the larger the L_max_, the more predictable the path). It is important to note that in our application we cannot interpret this measure in the rigorous dynamical sense and therefore we use it in terms of predictability.

The second measure in our analysis is recurrence-based determinism (DET) [10,13]. This measure gives the probability that a recurrence (of any state) will further recur. A further recurrence corresponds to segments of the trajectory that run parallel for some time, i.e. where the system evolves along the same path as in a previous occurrence. This can be derived from an RP and is the fraction of recurrence points forming diagonal line structures in the RP. In general, stochastic systems have RPs almost entirely made up of single points, whereas periodic systems have RPs with very long (theoretically infinitely long, only restricted by the limited size of the RP), continuous diagonal line structures. Chaotic systems also have continuous diagonal line structures, though these are shorter than in periodic systems. The term “determinism” is to be understood from this point of view, i.e., it is not deterministic in the mathematical sense, but meaning that the dynamics is not simply stochastic.

RQA is a powerful method for analyzing nonlinear dynamic systems. It has been extensively used in science and medicine [10,14,15], e.g., to analyze transitions in oil–water flows [16], to detect climate regime shifts [17] and event-related potentials in cognitive science [18], and to classify cardiovascular data [19]. Recent extensions of the method allow multi-scale recurrence analysis [20] and coupling/synchronization investigations [21]. A discussion of the potentials of RQA can be found in [22–23].

#### The AOF hypothesis

**Fig 3:**
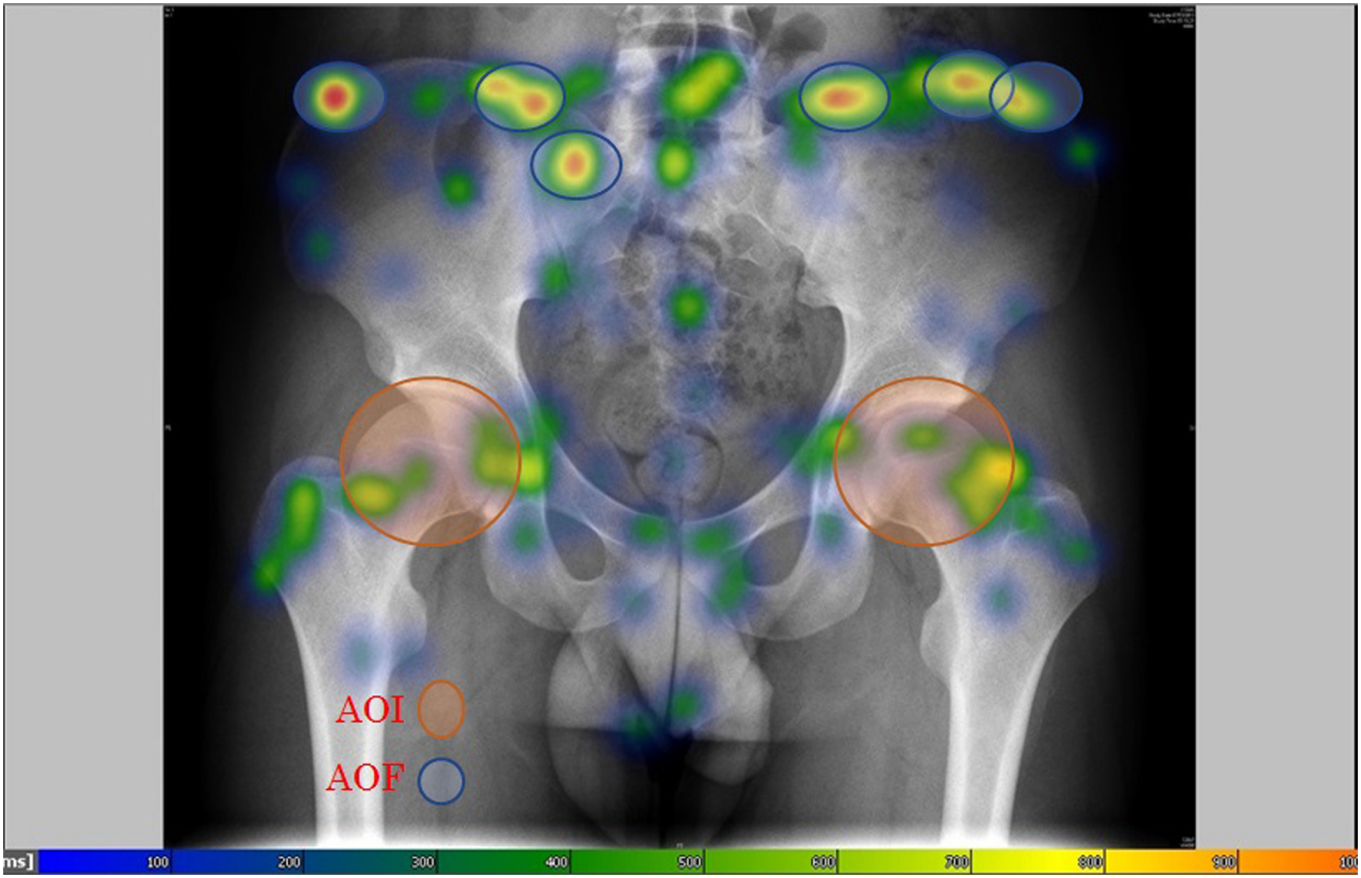
AOI and AOF example. A heat map. The AOFs are represented by the blue lines encircling the red areas of the heat map. The AOIs are represented by the orange circles reflecting the pathological areas evidencing FAI syndrome.

AOIs were collected from the post-operative diagnosis of pathological areas and, as explained above, were defined by experts. AOFs were identified through heat map analysis depicting the most frequently visited areas as determined through the time of the subjects’ eye fixations. Fig 3 presents an example of such a heat map; the AOFs are represented by the red areas. To test the AOF hypothesis we used the following dependent variables: AOF number, correct AOF (AOF in AOI), and percentage of correct AOF (i.e. correct AOF divided by total AOF).

#### The comparison hypothesis

Comparisons between symmetric areas of the pelvis were counted manually in the scan path video by tracing the movement of the eyes between the symmetric regions. To test the comparison hypothesis, we used the following dependent variables: number of comparisons and time to first comparison.

## Results

To compare the experts and the residents, given the small number of participants, we used the Mann–Whitney U test with a Monte Carlo simulation of 10,000 samples.

### Replication of previous findings

First, we examined whether our results replicate those of previous studies by comparing the experts and residents on five measures used in previous studies: total dwell time, fixation number, fixation duration, scan path length, and saccadic amplitude. To avoid the inflation of type I error as a result of multiple comparisons, the alpha level of the replication phase was set to 0.01 (i.e. 0.05/5). In line with previous findings it was found that:

1. the experts spent significantly less time per slide before reaching a diagnosis (M = 30 sec. vs. M = 37 sec. respectively, U = 34.0, p = 0.000);
2. the experts made significantly fewer fixations (M = 86 vs. M = 110 respectively, U = 6.0, p = 0.000);
3. the experts made significantly shorter fixations (M = 0.24 sec. vs. M = 0.27 sec. respectively, U = 6.0, p = 0.000);
4. the experts’ saccadic eye movement traveled a significantly greater distance (M = 212 pixels per fixation vs. M = 177 pixels per fixation respectively, U = 21.0, p = 0.000);
5. the experts’ saccadic amplitude was significantly longer (expert M = 6 per fixation vs. M = 4 per fixation, U = 9.0, p = 0.000), which means that the experts’ eyes traveled longer distances.

### The scan path hypothesis

To analyze the dynamic patterns in which radiographs are scanned, as previously described, we segmented each radiograph into nine AOIs and measured the fixations on these areas along a time line. The scan path of each participant was represented as a time series of the visited areas and was analyzed using the RQA measures of L_max_ and DET.

First, and to test our research hypothesis, we compared experts’ and novices’ time series by using the measure of L_max_. Inspecting the RPs, we can see that the longest diagonal line of the expert is shorter than the longest diagonal line of the resident (Fig 2c and 2d). It was found that the experts’ dynamic scan path scored significantly lower on the L_max_ measure (M = 7.8 vs. M = 9.3 respectively, U = 55.5, p = 0.017), meaning that the experts’ dynamics is less predictable than those of the residents.

Next, we calculated the DET measure of complexity and found that its values were around 0.62 (median), both for novices and experts. We tested this result against the null hypothesis of non-deterministic and stochastic behavior by using 500 shuffled surrogates (for all 210 test cases). We found only five cases with p > 0.1; for the remaining cases p < 0.02 (majority is p ~ 0.000), supporting the conclusion that the dynamics of the participants’ scan path was not stochastic but deterministic. When comparing novices with experts, the DET values were not found to be significantly different (p = 0.41). In sum, we found that both experts and novices follow a deterministic scan path, but the experts’ scan path was less predictable, as indicated by the L_max_ measure

### The AOI hypothesis

**Fig 4:**
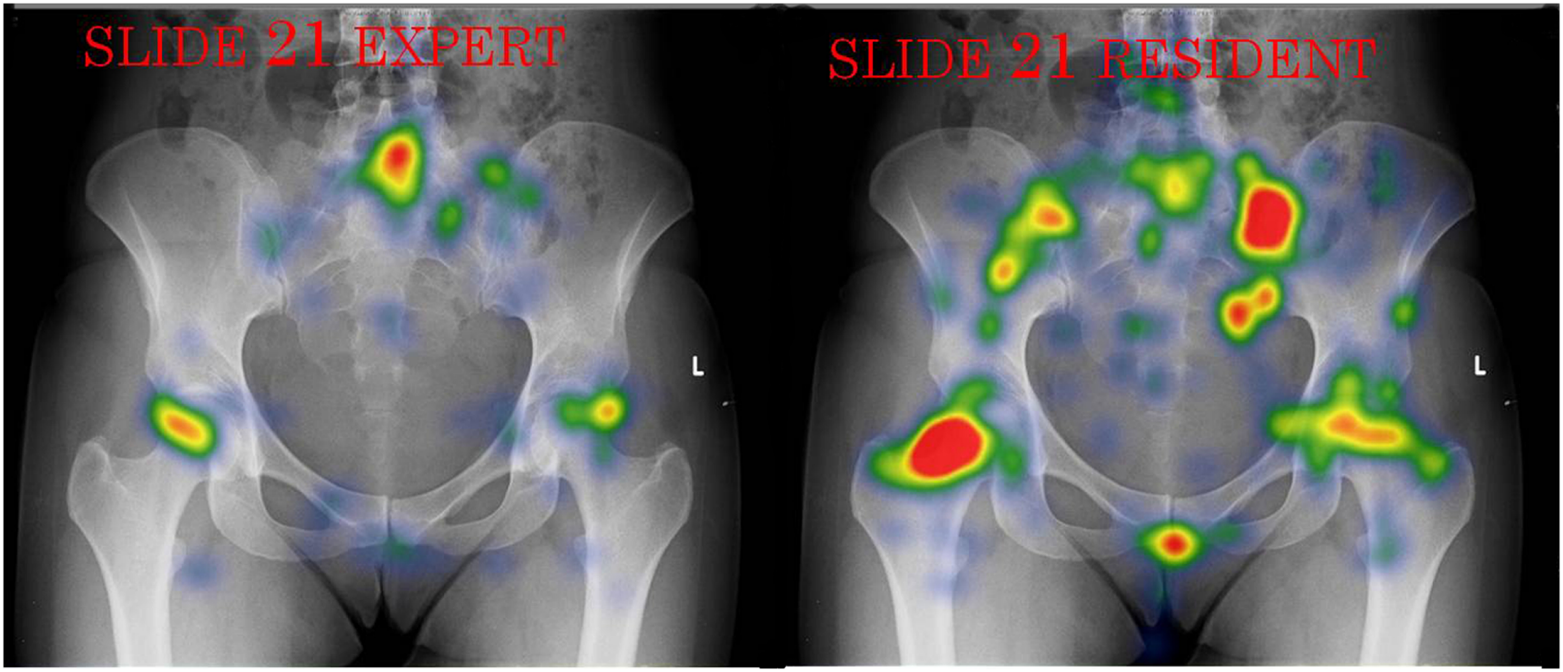
An heatmap example of an expert vs. a resident. this figure represents a comparison between two heat maps (those of the experts and the residents).This figure contains 3 pathologic areas, the hip joints and suspected Spina Bifida. This comparison emphasizes the differences in experts’ and residents’ attention to AOFs and their relation to AOIs.

**Fig 5:**
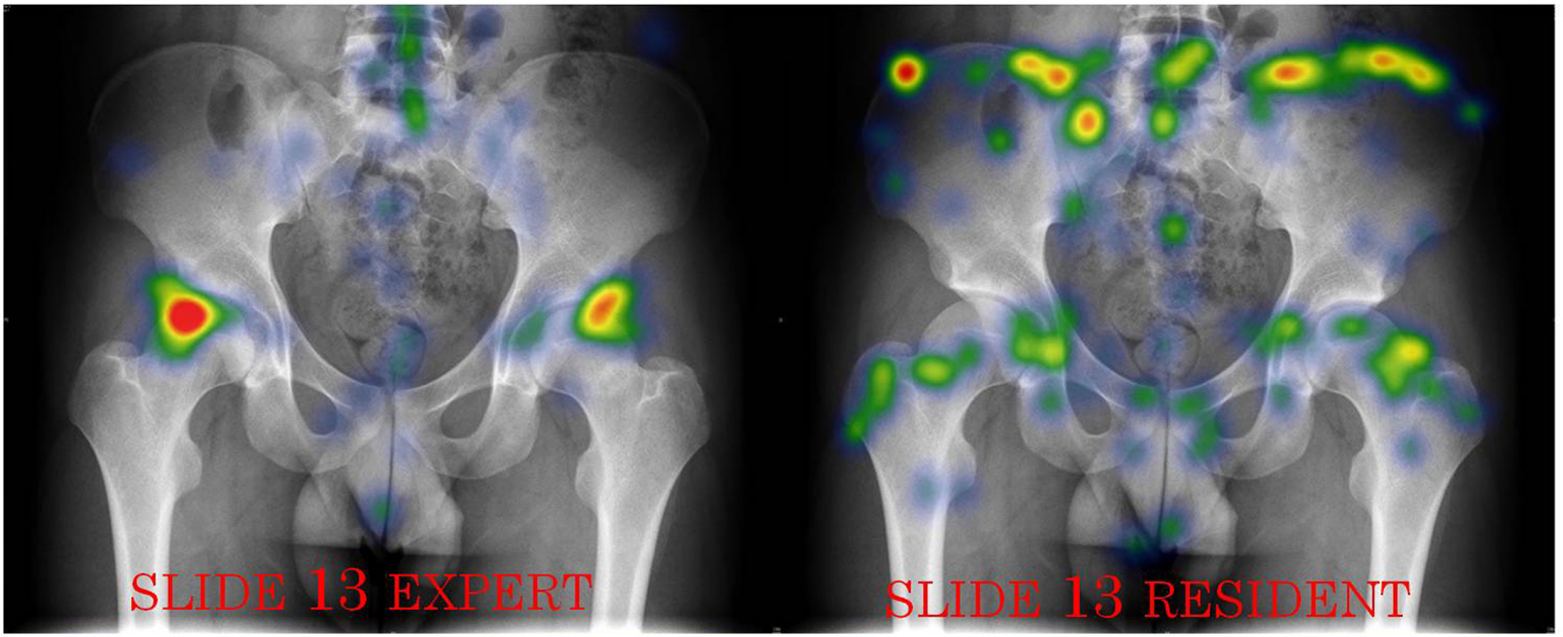
A second heatmap example of an expert vs. a resident. this figure represents a comparison between two heat maps (those of the experts and the residents).This figure contains only 2 pathologic areas (the hip joints). This comparison emphasizes the differences in experts’ and residents’ attention to AOFs and their relation to AOIs.

It was found that experts had significantly more focus areas in the pathological areas (i.e. AOIs) (M = 1.7 vs. M = 1.2 respectively, U = 50.0, p = 0.009). Although experts made fewer fixations, they had more AOFs per fixation (M = 0.03, vs. M = 0.026 respectively, U = 66.0, p = 0.024). This finding means that experts do not’ “waste” their fixations and rather focus most of their fixations on AOIs. Indeed, it was found that experts had 20% more AOFs in the pathological area as measured by the percentage of correct AOFs (M = 65% vs. M = 45% respectively, U = 34.5, p = 0.000); see Figs 4 and 5.

### The comparison hypothesis

As we hypothesized, it took the experts significantly less time to make their first comparison (M = 4.2 sec. vs. M = 7.6 sec. respectively, U = 27.0, p = 0.000). In addition, experts made more comparisons per slide (M = 7.1, vs. M = 5.8 respectively, U = 56.5, p = 0.018).

## Discussion

Clinical diagnosis is an integral part of medicine and is an “acquired talent” with a direct relationship to experience, as has been demonstrated in the current study. In this context, learning to be an expert is far more complex than what has been described for years in the cognitive literature dealing with the nature of expertise. Better understanding the nature of expertise may help us to develop training protocols for the non-expert physician.

One of our aims was to identify the qualitative differences between experts and non-experts in order to utilize this finding in the medical education of novice health care providers, such as medical residents and even students.

Previous studies of expertise that used eye tracking usually analyzed simple measures. However, in the current study, we found that experts also differ from residents in terms of more complex measures. First, by using a methodology to analyze dynamic patterns of scanning, we found that the experts’ scan path has a more complex and less predictable signature, although both groups, experts and novices, express a deterministic dynamic in scanning a radiograph. By losing our commitment to the rather technical meaning of the term “chaotic,” we may speculate that the less predictable dynamics exhibited by the experts mean that the scan path of the experts is highly sensitive to initial conditions, is deterministic, and is unpredictable. What this means is that the experts’ scan path is highly sensitive to the first areas of the radiograph on which they focus; metaphorically, like a duel in an old Western, the first shot is crucial. The deterministic aspect of their "chaotic" pattern means that the experts’ scan path is highly structured and that they move between the areas in an ordered way. However, their pattern is, nevertheless, less predictable, meaning that it cannot be easily imitated to gain expertise.

When we compared physicians’ AOFs to the AOIs, we found a pattern in which experts had more AOFs in the relevant AOIs and were thus more efficient than non-experts. By taking into account the fact that experts had a greater ratio of focus areas to fixation numbers and at the same time had higher percentages of correct AOFs, we can hypothesize that expertise involves the ability to use fewer fixations to identify areas that are prone to injuries. Therefore, we may conclude that experts are not just faster “machines” but better in recognizing the relevant target areas.

It has been argued [24] that experts are guided by the retrieval of schemes that effectively guide their practice. Schemes, such as those for scan paths, may be an important tool in the education of residents. Indeed, it has been found [25] that exposure to various scan paths may improve diagnostic performance. Therefore, a practical implication of our study may be the design of a “diagnostic simulator” in which residents are trained to scan a radiograph along similar paths to those used by experts. This proposal is grounded in an evidence-based approach to the training of practitioners [26]. Such an approach may be used to guide the practical applications of our study, and probably through the use of artificial intelligence tools. For instance, given the dynamic patterns found in our study, we may automatically produce various scan paths to train residents. The resident may be presented with various radiographs and be asked to follow certain scan paths. While scanning the radiograph, his/her eyes may be monitored through an eye tracker that may feed back to the resident when the gaze slips away from the relevant zones. In sum, the simulator may be a combination of an artificial intelligence kind of system that produces scan paths, an eye tracker that monitors the resident’s eyes, and a feedback system that provides the subject with relevant corrections. To the best of our knowledge such a system does not’ exist, at least in our limited context of FAI.

The abovementioned results and explanations should be qualified given the study’s limitations. The sample size was small but we tried to compensate for this fact by using a nonparametric test that included a simulation of 10,000 samples. In addition, we used only AP pelvic radiographs, thus limiting the validity of our conclusions to this specific pathology. In addition, FAI syndrome is a relatively new concept and as such there is relatively limited experience in its diagnosis among orthopedists. Given these qualifications, our study presents primary results that may advance the understanding of expertise and that may guide future studies and applications.

## Appendix

A recurrence plot is a binary matrix R(i,j) representing the pairwise test of all values of a state space trajectory (or time series, as in our study), whether their distance is smaller than or equal to some threshold ε, i.e. R(i,j) = 1 iff ||x(i)–x(j)|| ≦ ε, else R(i,j) = 0. The threshold ε defines what is considered to be a recurrence. There are several approaches to selecting ε (11). In our study, we analyzed discrete values and, therefore, consider identity for the recurrence: R(i,j) = 1 iff x(i) ≡ x(j). Therefore, we did not select a threshold. Moreover, we only considered time series and did not embed (in order to reconstruct a high-dimensional state space). Therefore, we did not select further parameters such as embedding dimension or delay.

## Supporting Information

S1 Data Experiment data and result files. The zip files includes 15 main radiographs used in the experiment (in main radiograph folder), all the gathered data (in the eyetracking data folder) and all the result files used for the statistic analysis (hypothesis 1 & 2, newstat, rqanew and rqanew2).
(ZIP)
