# Supplementary figures and images for: Dynamic Patterns of Expertise: The Case of Orthopedic Medical Diagnosis
Source: PLoS One. 2016 Jul 14;11(7):e0158820. doi: 10.1371/journal.pone.0158820 (PMC4945032; doi:10.1371/journal.pone.0158820)

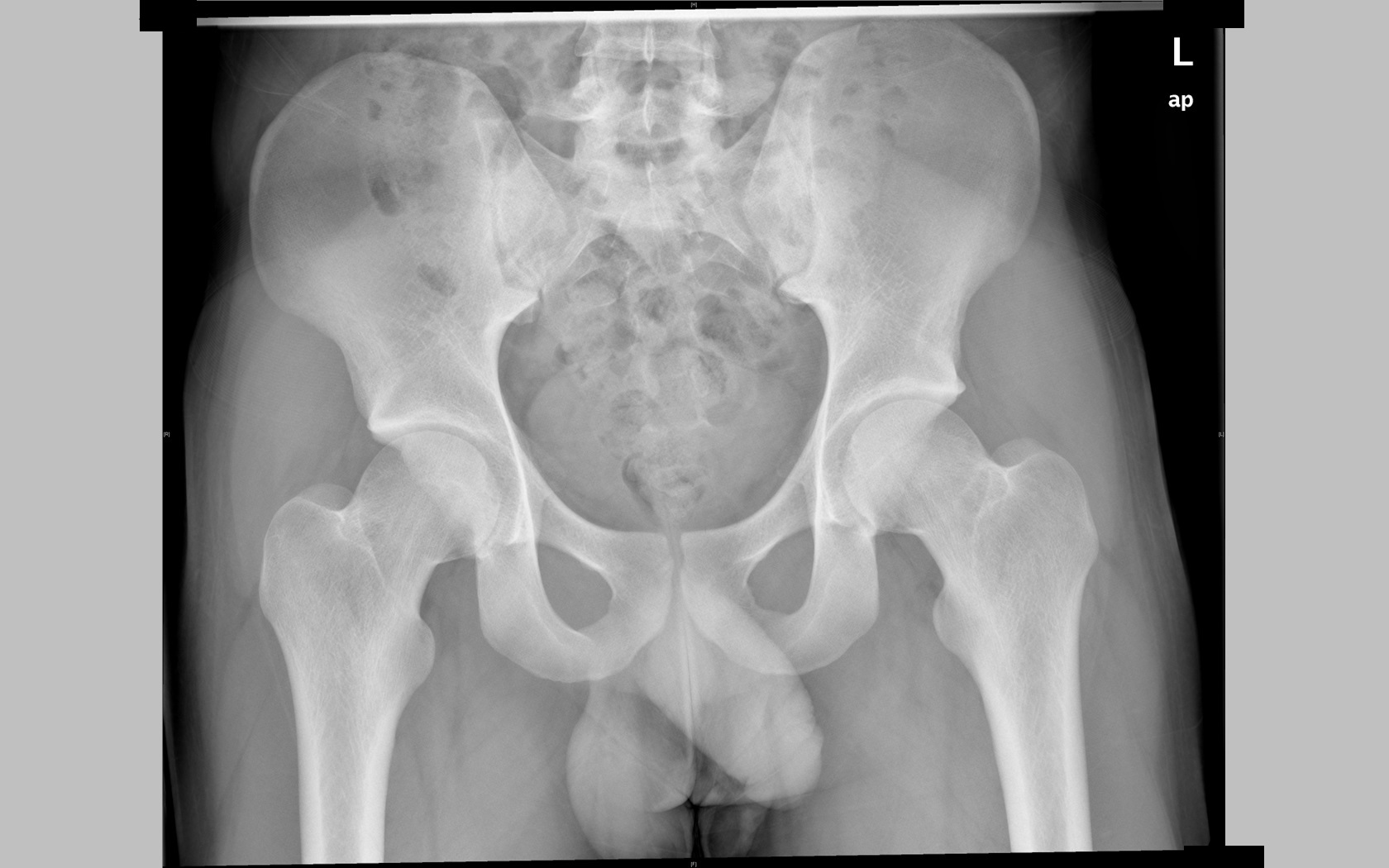

Supplement: S1 Data — The zip files includes 15 main radiographs used in the experiment (in main radiograph folder), all the gathered data (in the eyetracking data folder) and all the result files used for the statistic analysis (hypothesis 1 & 2, newstat, rqanew and rqanew2). (ZIP) [file pone.0158820.s001.zip › fai data/main radiographs/N1.jpg]

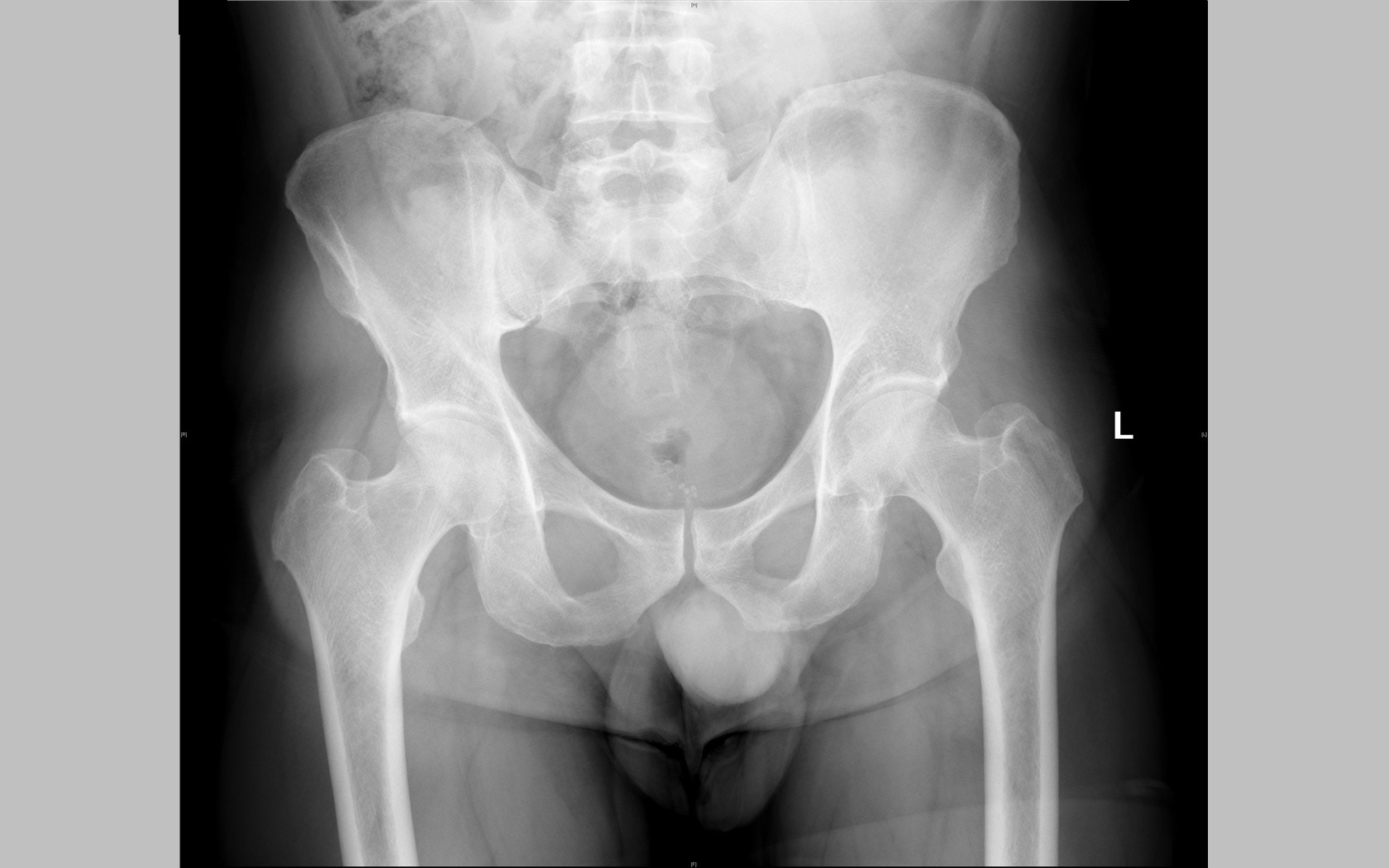

Supplement: S1 Data — The zip files includes 15 main radiographs used in the experiment (in main radiograph folder), all the gathered data (in the eyetracking data folder) and all the result files used for the statistic analysis (hypothesis 1 & 2, newstat, rqanew and rqanew2). (ZIP) [file pone.0158820.s001.zip › fai data/main radiographs/N2.jpg]

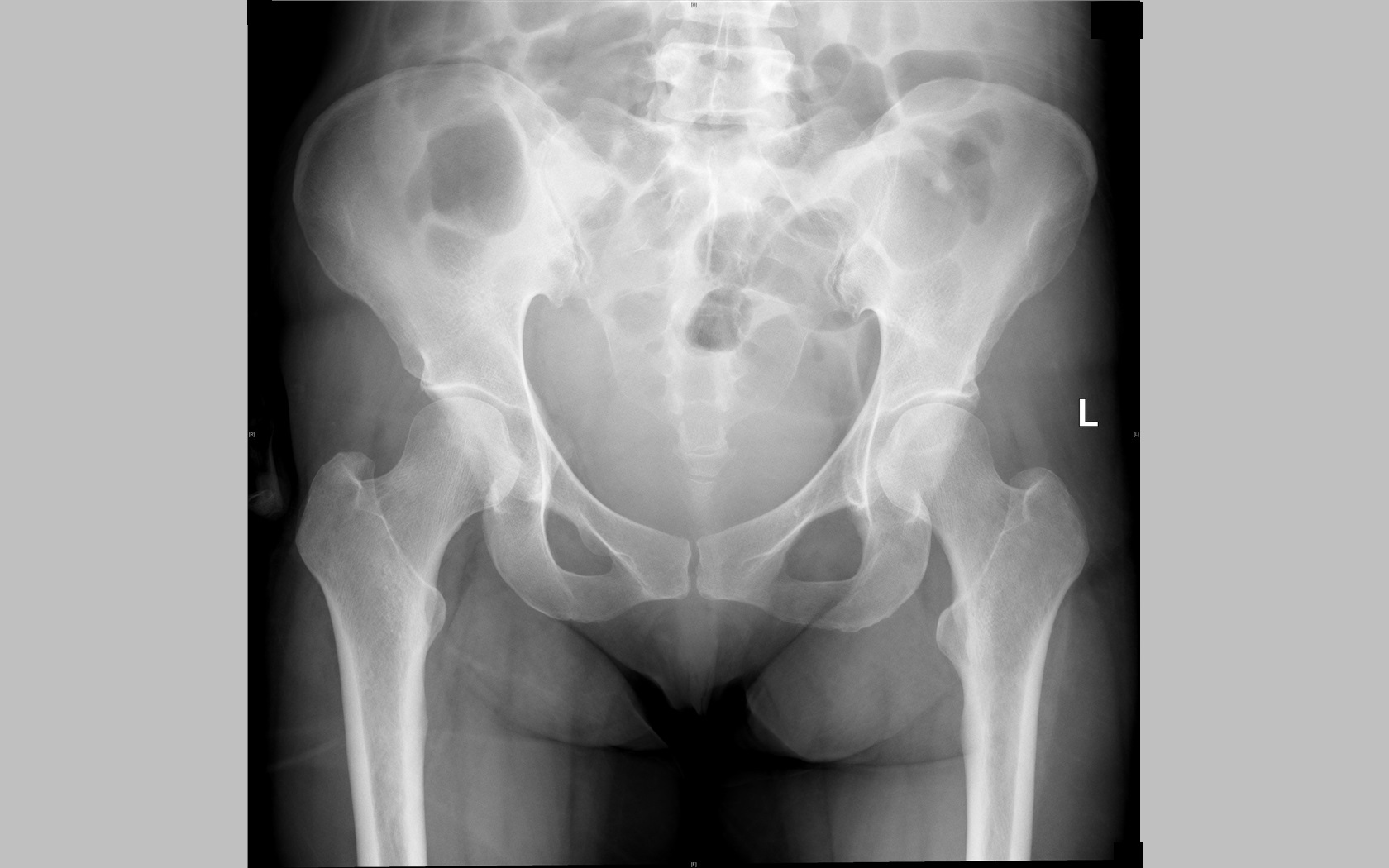

Supplement: S1 Data — The zip files includes 15 main radiographs used in the experiment (in main radiograph folder), all the gathered data (in the eyetracking data folder) and all the result files used for the statistic analysis (hypothesis 1 & 2, newstat, rqanew and rqanew2). (ZIP) [file pone.0158820.s001.zip › fai data/main radiographs/N5.jpg]

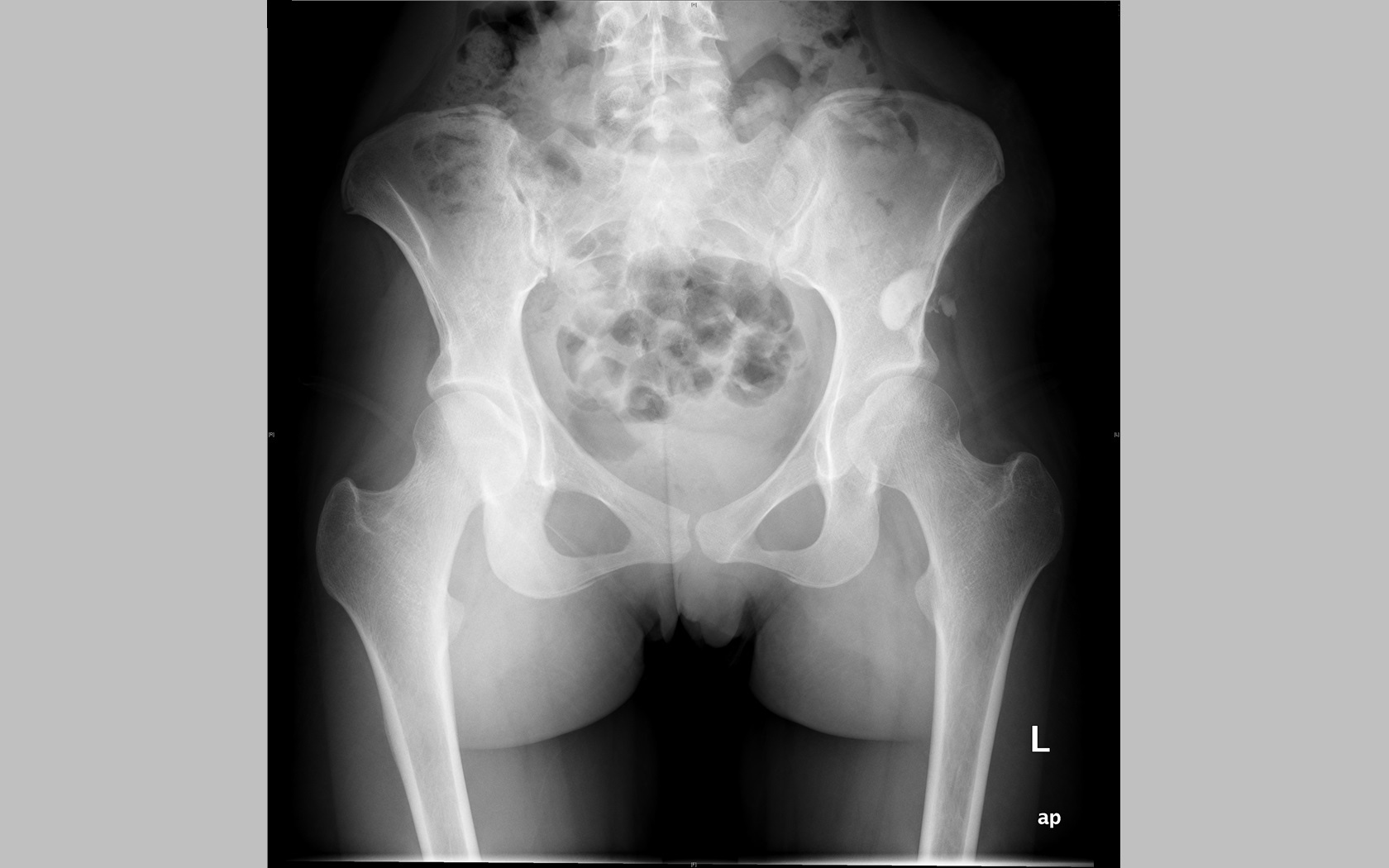

Supplement: S1 Data — The zip files includes 15 main radiographs used in the experiment (in main radiograph folder), all the gathered data (in the eyetracking data folder) and all the result files used for the statistic analysis (hypothesis 1 & 2, newstat, rqanew and rqanew2). (ZIP) [file pone.0158820.s001.zip › fai data/main radiographs/N6.jpg]

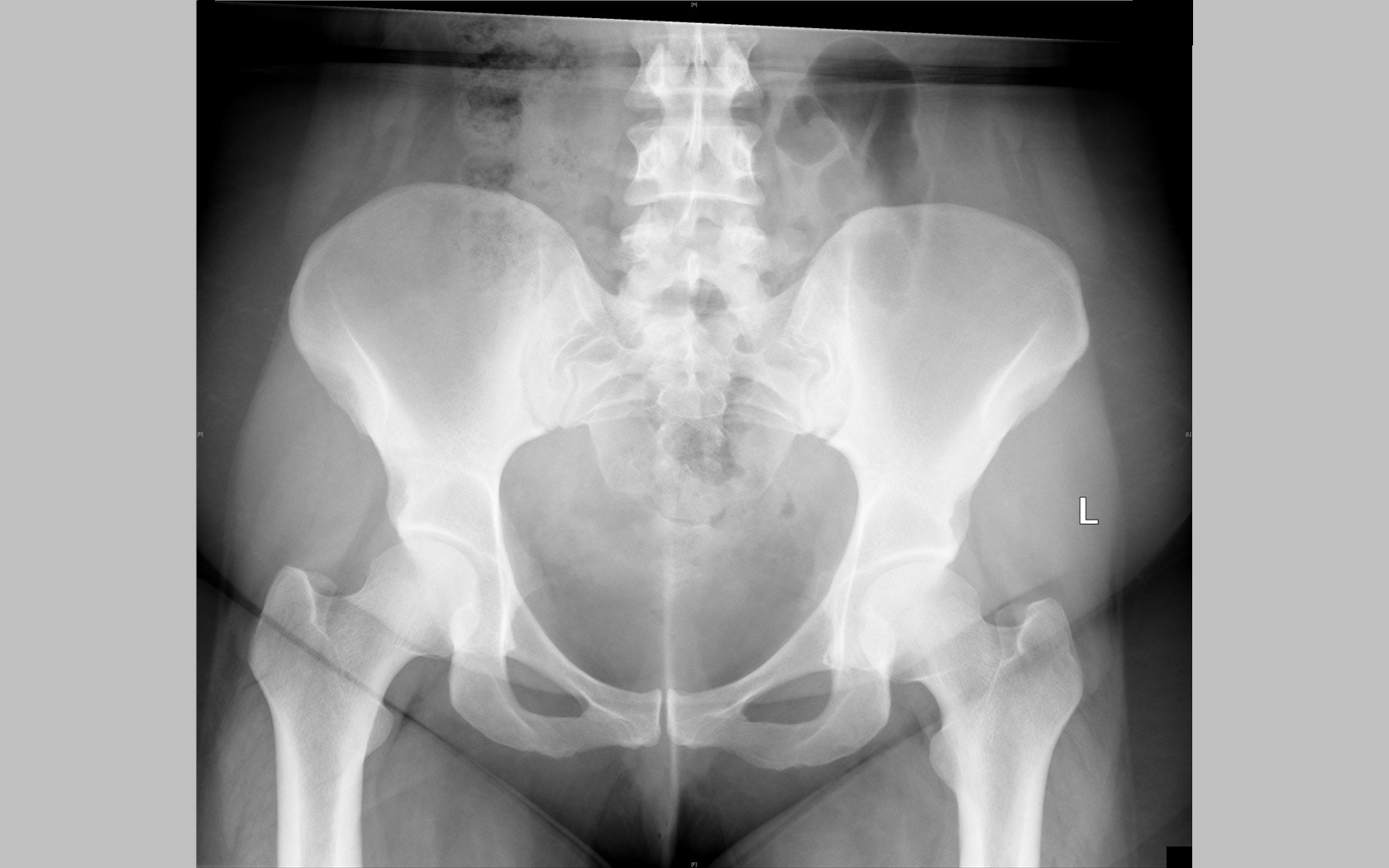

Supplement: S1 Data — The zip files includes 15 main radiographs used in the experiment (in main radiograph folder), all the gathered data (in the eyetracking data folder) and all the result files used for the statistic analysis (hypothesis 1 & 2, newstat, rqanew and rqanew2). (ZIP) [file pone.0158820.s001.zip › fai data/main radiographs/N9.jpg]

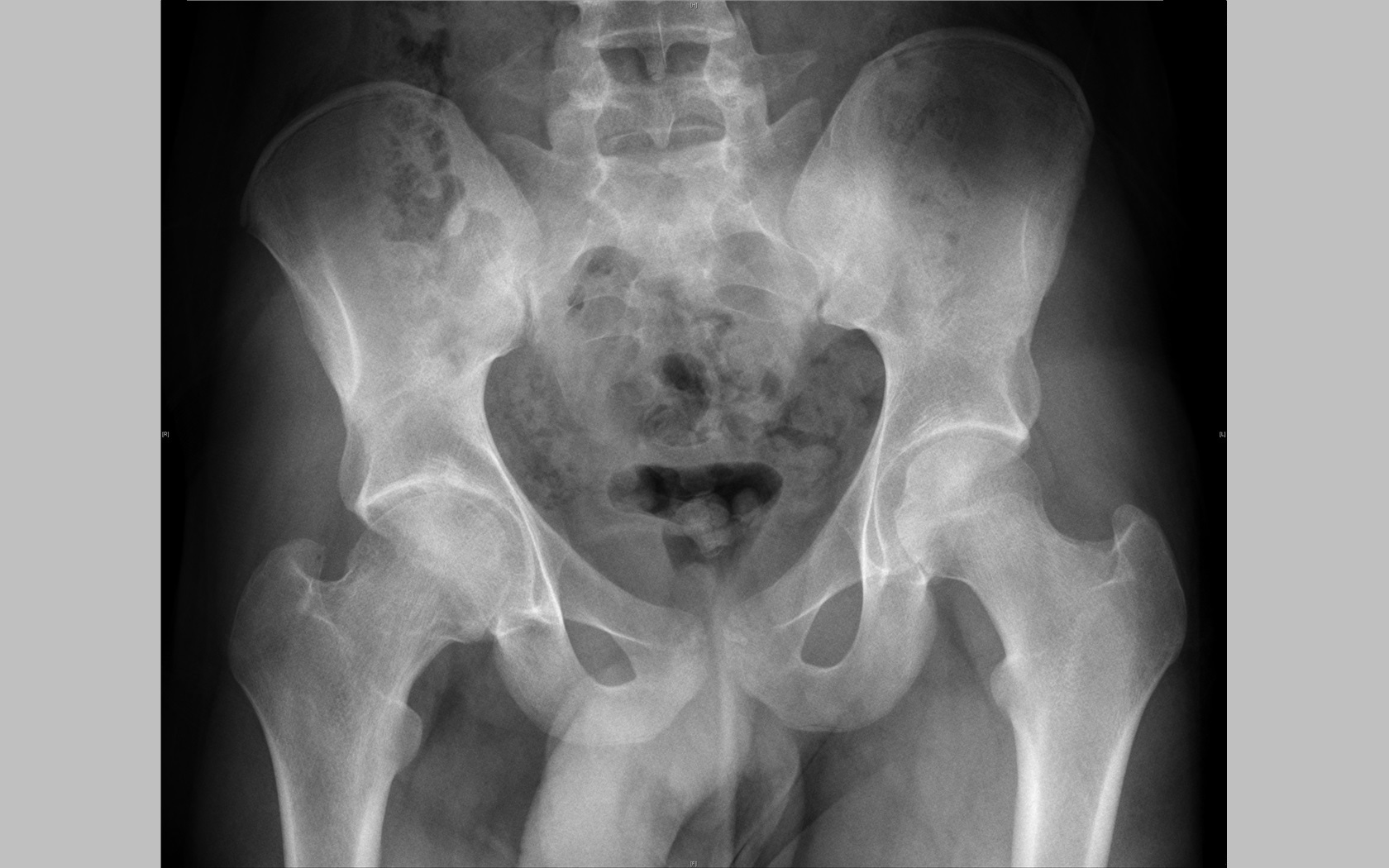

Supplement: S1 Data — The zip files includes 15 main radiographs used in the experiment (in main radiograph folder), all the gathered data (in the eyetracking data folder) and all the result files used for the statistic analysis (hypothesis 1 & 2, newstat, rqanew and rqanew2). (ZIP) [file pone.0158820.s001.zip › fai data/main radiographs/P1.jpg]

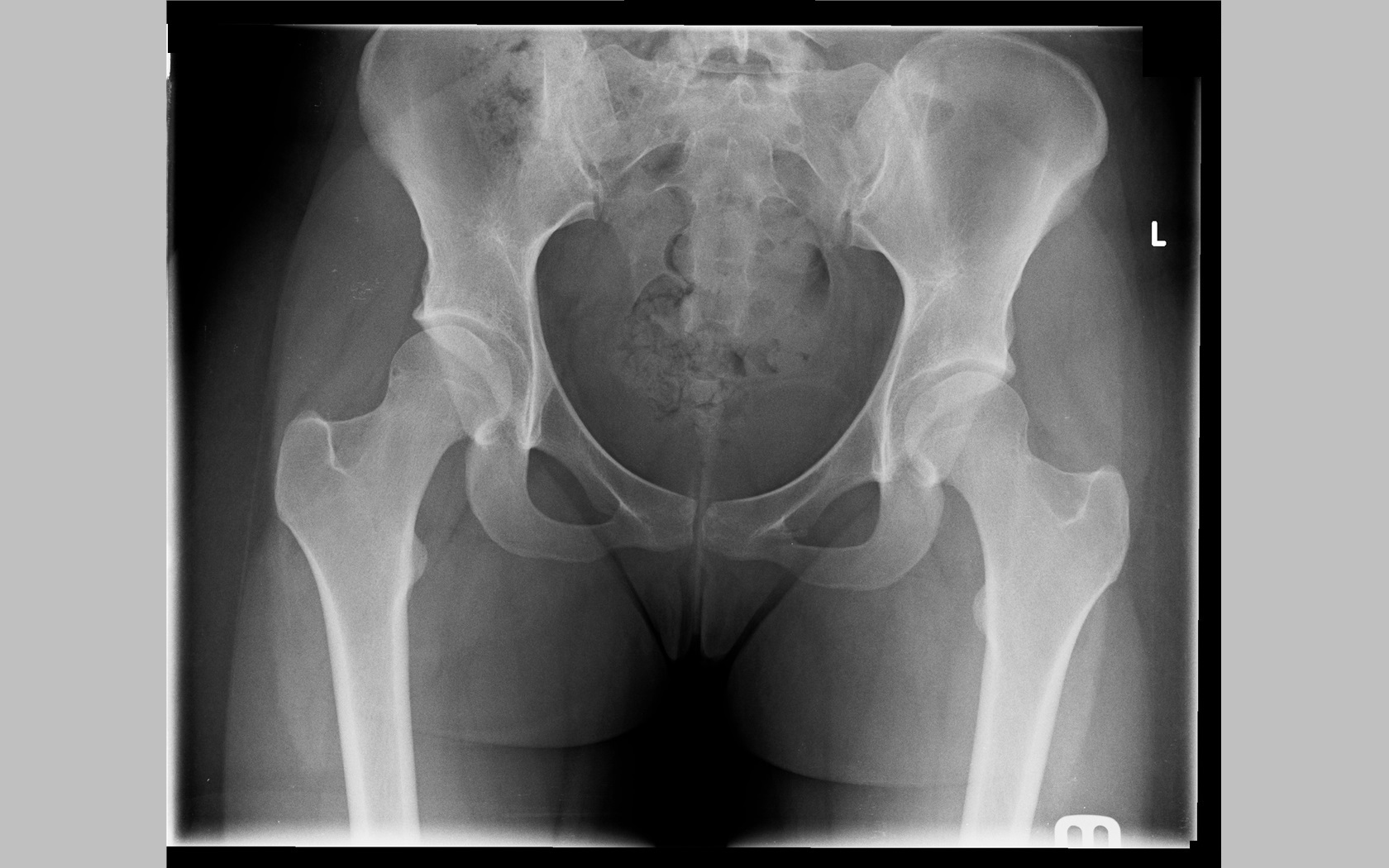

Supplement: S1 Data — The zip files includes 15 main radiographs used in the experiment (in main radiograph folder), all the gathered data (in the eyetracking data folder) and all the result files used for the statistic analysis (hypothesis 1 & 2, newstat, rqanew and rqanew2). (ZIP) [file pone.0158820.s001.zip › fai data/main radiographs/P10.jpg]

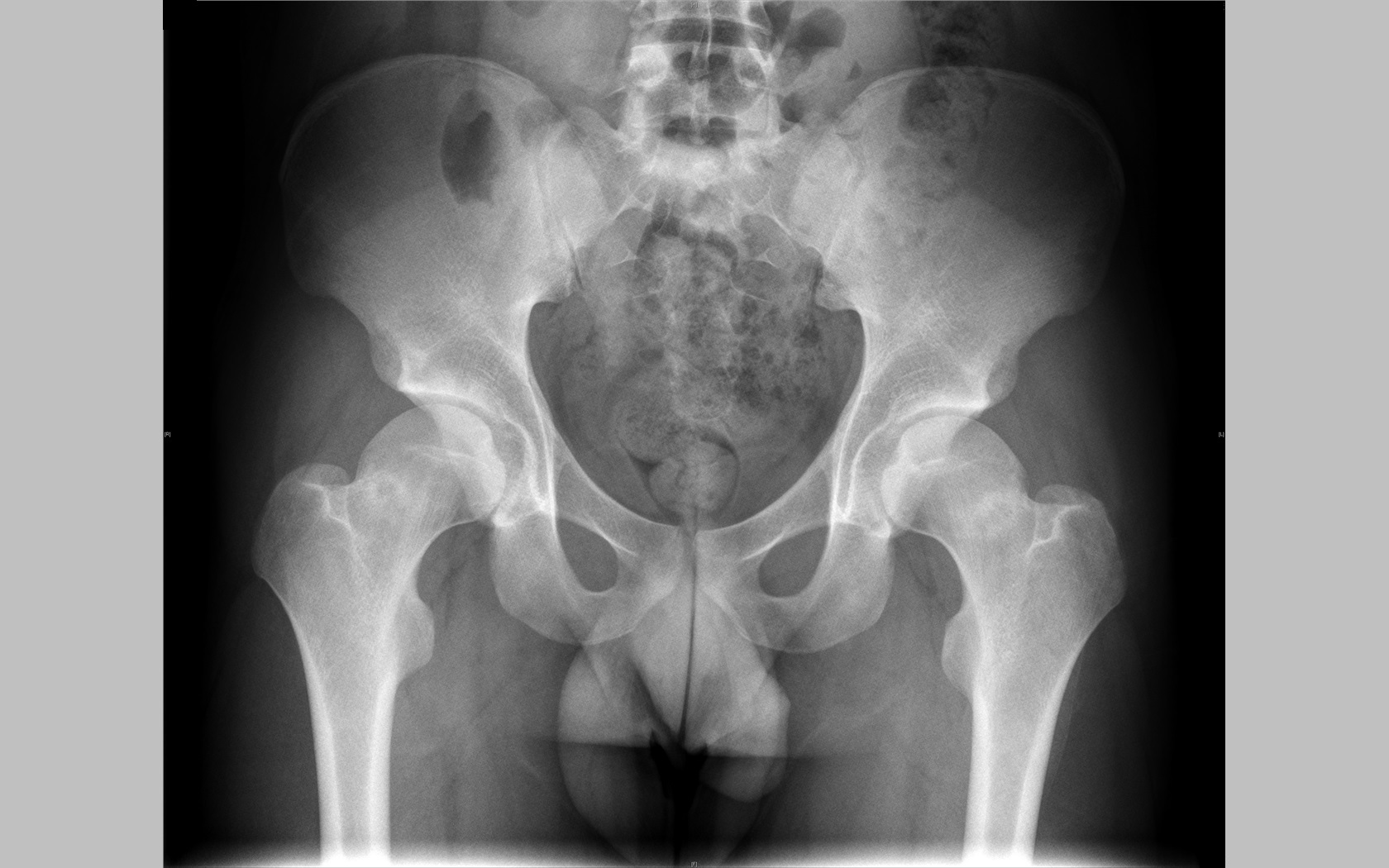

Supplement: S1 Data — The zip files includes 15 main radiographs used in the experiment (in main radiograph folder), all the gathered data (in the eyetracking data folder) and all the result files used for the statistic analysis (hypothesis 1 & 2, newstat, rqanew and rqanew2). (ZIP) [file pone.0158820.s001.zip › fai data/main radiographs/P13.jpg]

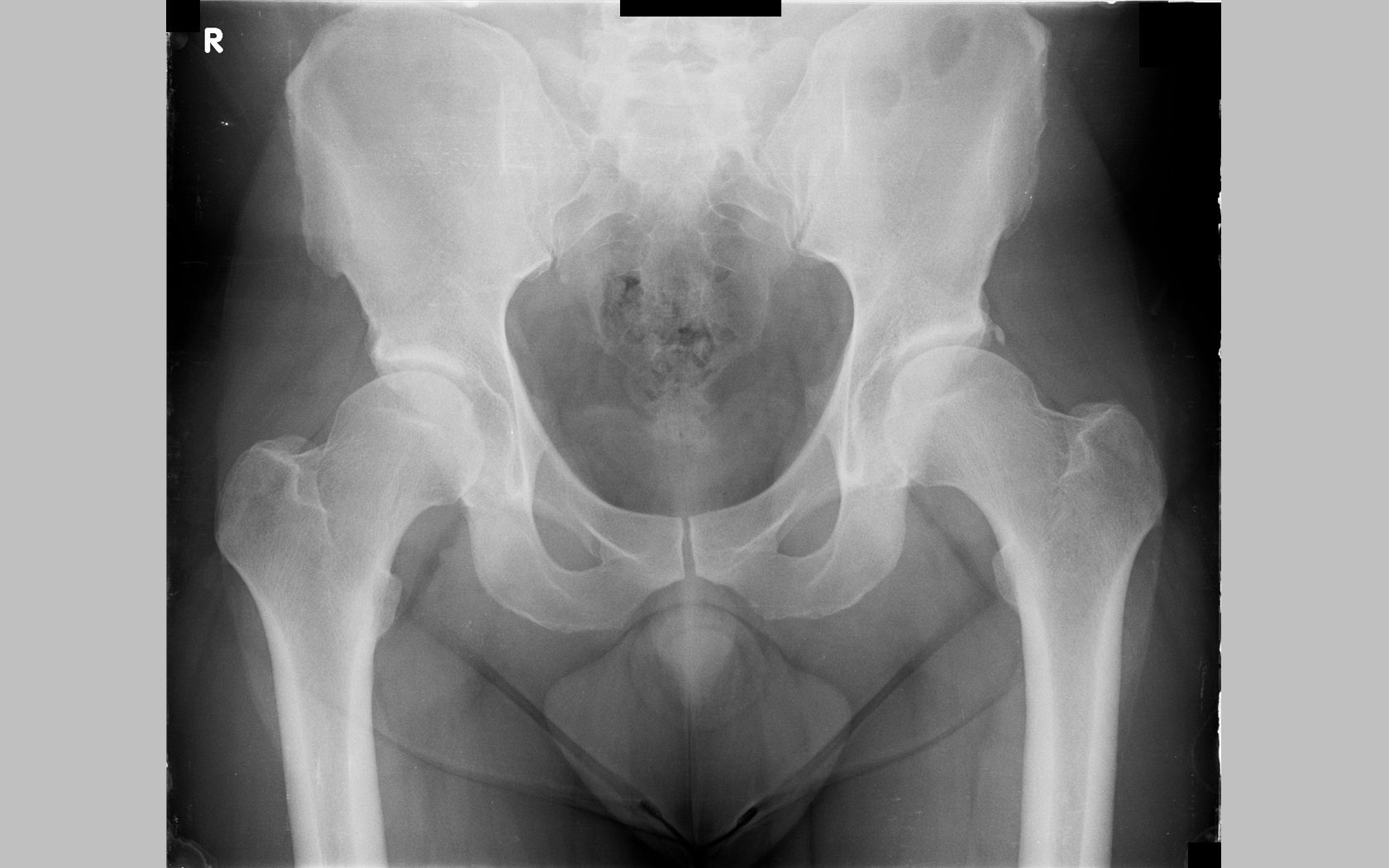

Supplement: S1 Data — The zip files includes 15 main radiographs used in the experiment (in main radiograph folder), all the gathered data (in the eyetracking data folder) and all the result files used for the statistic analysis (hypothesis 1 & 2, newstat, rqanew and rqanew2). (ZIP) [file pone.0158820.s001.zip › fai data/main radiographs/P20.jpg]

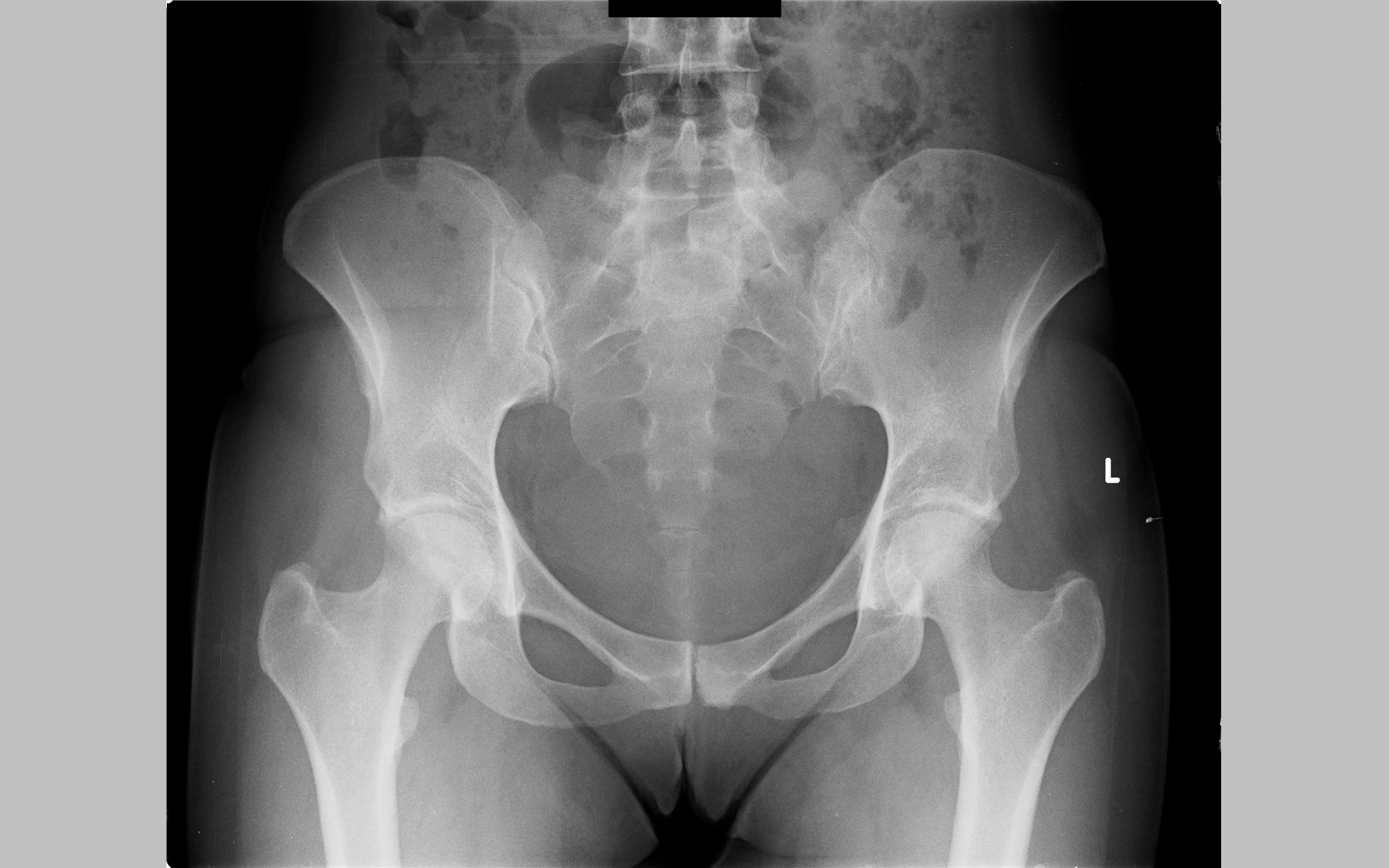

Supplement: S1 Data — The zip files includes 15 main radiographs used in the experiment (in main radiograph folder), all the gathered data (in the eyetracking data folder) and all the result files used for the statistic analysis (hypothesis 1 & 2, newstat, rqanew and rqanew2). (ZIP) [file pone.0158820.s001.zip › fai data/main radiographs/P21.jpg]

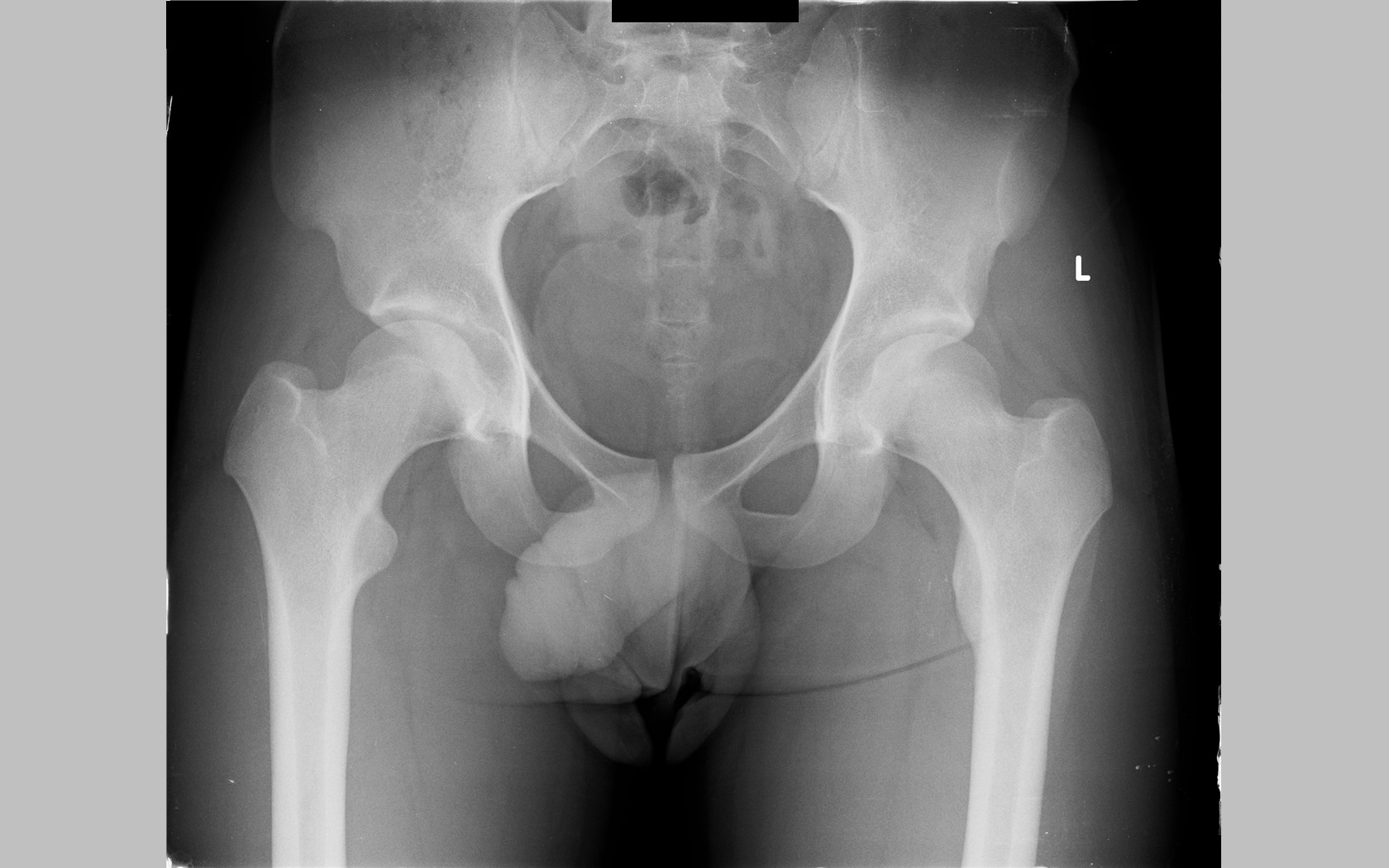

Supplement: S1 Data — The zip files includes 15 main radiographs used in the experiment (in main radiograph folder), all the gathered data (in the eyetracking data folder) and all the result files used for the statistic analysis (hypothesis 1 & 2, newstat, rqanew and rqanew2). (ZIP) [file pone.0158820.s001.zip › fai data/main radiographs/P22.jpg]

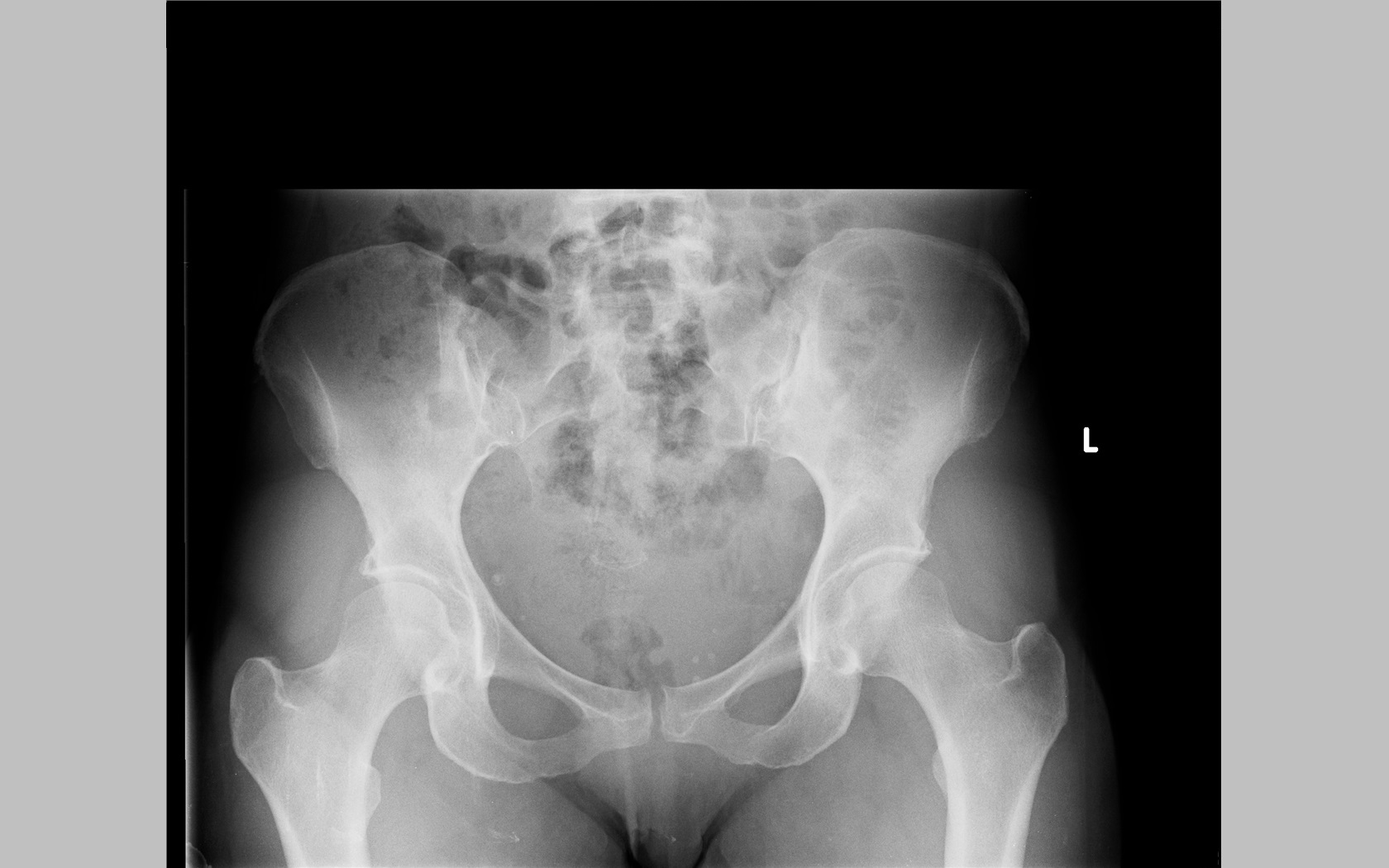

Supplement: S1 Data — The zip files includes 15 main radiographs used in the experiment (in main radiograph folder), all the gathered data (in the eyetracking data folder) and all the result files used for the statistic analysis (hypothesis 1 & 2, newstat, rqanew and rqanew2). (ZIP) [file pone.0158820.s001.zip › fai data/main radiographs/P26.jpg]

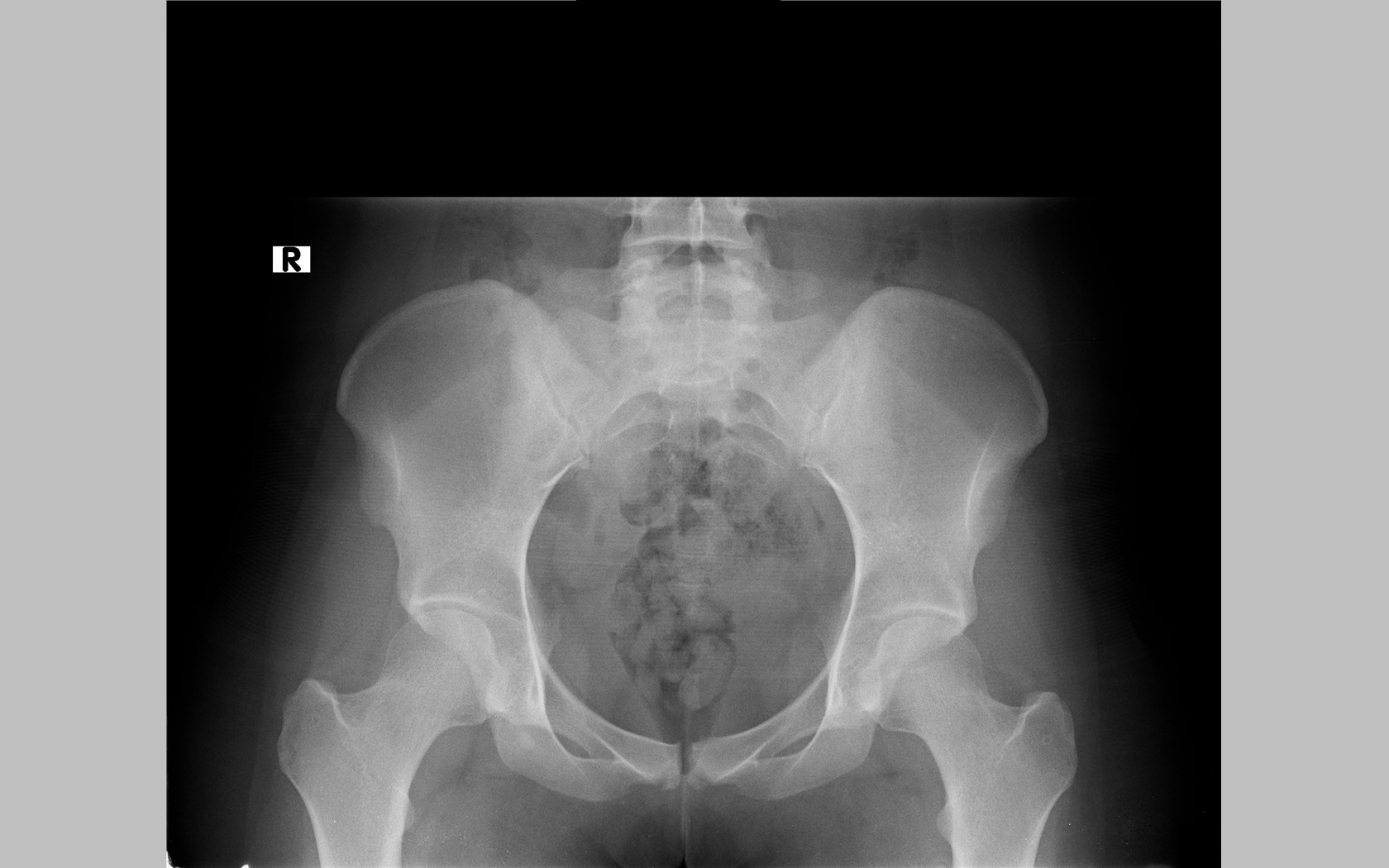

Supplement: S1 Data — The zip files includes 15 main radiographs used in the experiment (in main radiograph folder), all the gathered data (in the eyetracking data folder) and all the result files used for the statistic analysis (hypothesis 1 & 2, newstat, rqanew and rqanew2). (ZIP) [file pone.0158820.s001.zip › fai data/main radiographs/P27.jpg]

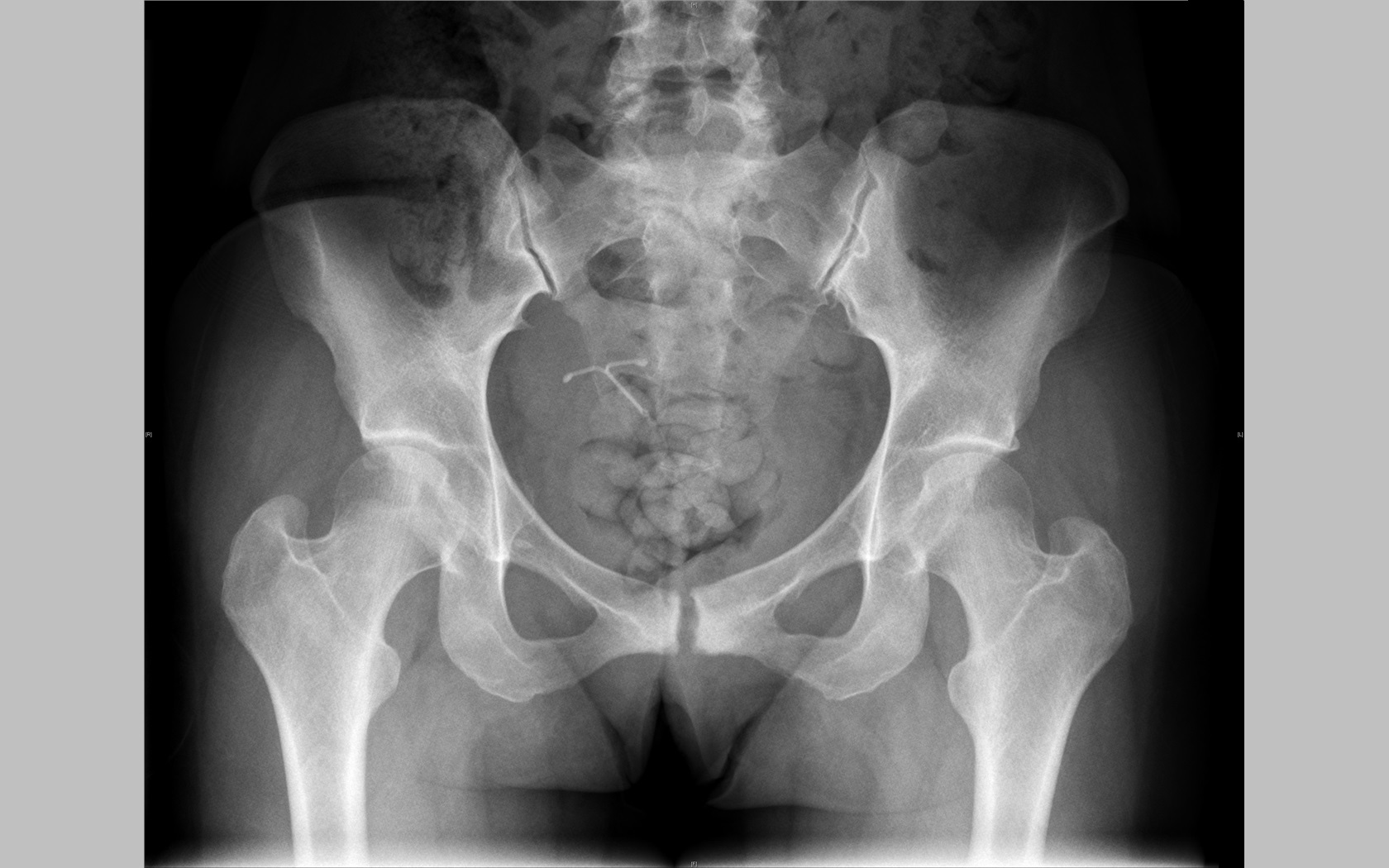

Supplement: S1 Data — The zip files includes 15 main radiographs used in the experiment (in main radiograph folder), all the gathered data (in the eyetracking data folder) and all the result files used for the statistic analysis (hypothesis 1 & 2, newstat, rqanew and rqanew2). (ZIP) [file pone.0158820.s001.zip › fai data/main radiographs/P6.jpg]

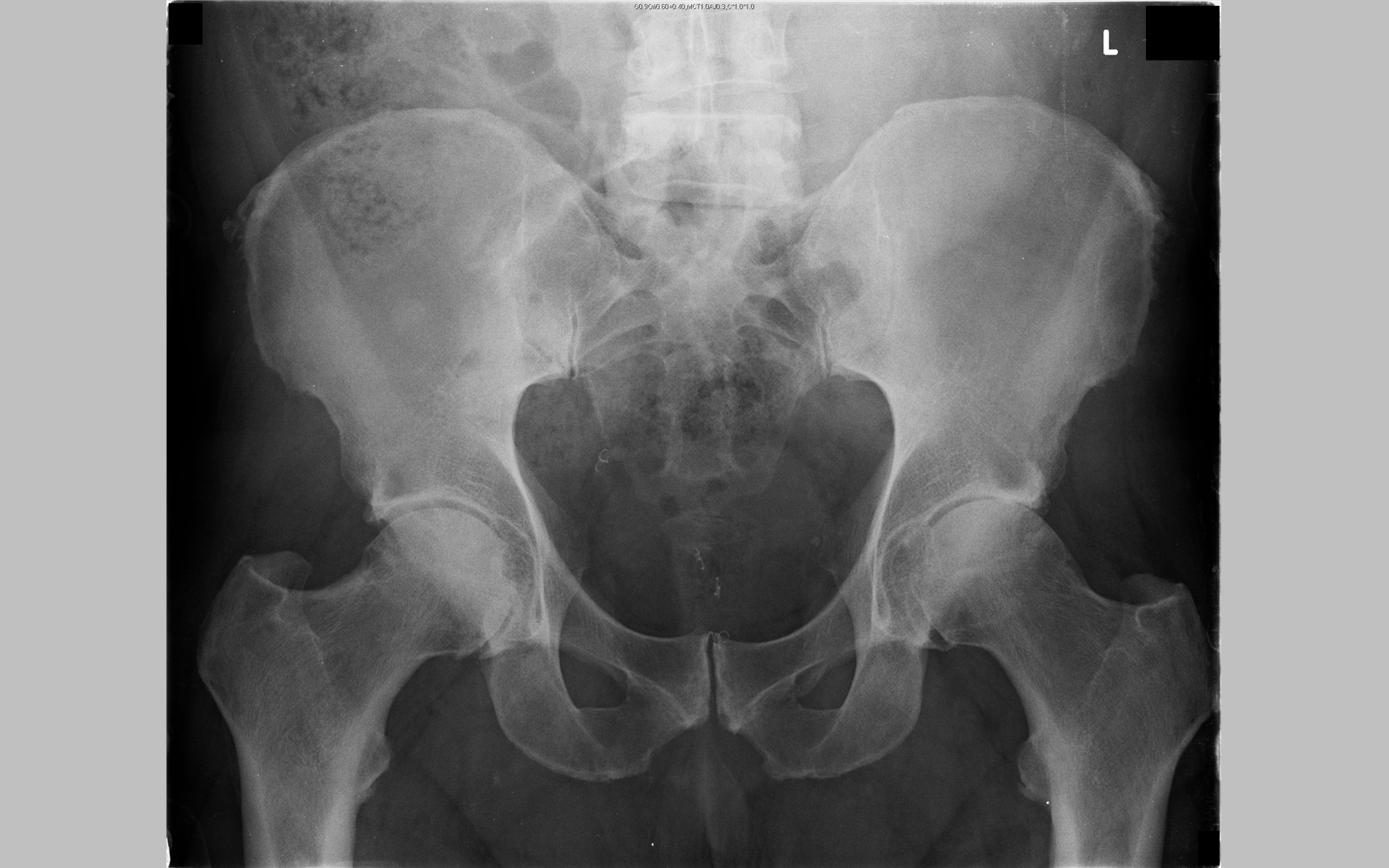

Supplement: S1 Data — The zip files includes 15 main radiographs used in the experiment (in main radiograph folder), all the gathered data (in the eyetracking data folder) and all the result files used for the statistic analysis (hypothesis 1 & 2, newstat, rqanew and rqanew2). (ZIP) [file pone.0158820.s001.zip › fai data/main radiographs/P7.jpg]
